# The feasibility of a combined approach including neuromodulation by tDCS and cognitive remediation for people with borderline personality disorder (BPD)

**DOI:** 10.1192/j.eurpsy.2024.377

**Published:** 2024-08-27

**Authors:** L. Cailhol

**Affiliations:** Psychiatry, Institut Universitaire de Santé Mentale de Montréal, Montreal, Canada

## Abstract

**Introduction:**

BPD is a common and severe mental health condition. Longitudinal studies related to BPD show a reduction of symptoms related to the disorder but very little improvement in functionality. The betterment of executive functions of people with BPD after psychotherapy is very limited. The efficacy of those treatments on functionality appears to be mild with a small effect size. Based on previous studies, transcranial direct current stimulation (tDCS) can be used to improve impulsivity and emotional instability in patients with BPD. Moreover, cognitive remediation focuses on reducing neuropsychological alterations by re-educating patients and apply specific strategies to aid them long term on certain daily functions like developing healthy habits, executive functions, problem solving, attention, working memory and cognition.

**Objectives:**

Our objective is to assess the feasibility and efficacy of the tDCS and cognitive remediation on BPD symptoms and functioning.

**Methods:**

The open study includes 10 daily sessions of tDCS for 2 weeks and 8 weekly group meetings for the cognitive remediation. Based on studies conducted on people with BPD, the settings for the tDCS are as follows; 20 minutes of continuous current at the intensity of 2mA and the electrodes are placed on specific stimulation sites related to impulsivity. To verify the effectiveness of the combination on the symptoms and evaluate the cognition and functionality of the patients, questionnaires at neuropsychological texts are conducted at the beginning of the study, after the tDCS, after the cognitive remediation and 3 months after the end of the study. The expected results of this study are that the combination of the two treatments will reduce the symptoms of BPD and improve executive functions compared to the treatment as usual or tDCS alone. This study would allow the implementation of an efficient and low-cost first-line treatment and a better functional progression of BPD patients.

**Results:**

The expected results of this study are that the combination of the two treatments will reduce the symptoms of BPD and improve executive functions compared to the treatment as usual or tDCS alone. This study would allow the implementation of an efficient and low-cost first-line treatment and a better functional progression of BPD patients.

**Conclusions:**

This study would allow the implementation of an efficient and low-cost first-line treatment and a better functional progression of BPD patients.

**Disclosure of Interest:**

None Declared

